# Takotsubo cardiomyopathy in two female patients: two case reports

**DOI:** 10.1186/1757-1626-1-325

**Published:** 2008-11-18

**Authors:** AJ Turley, RJ Graham, JA Hall

**Affiliations:** 1 Cardiothoracic Division, the James Cook University Hospital, Marton Road, Middlesbrough, TS4 3BW, UK

## Abstract

**Background:**

Tako-tsubo cardiomyopathy (idiopathic apical ballooning syndrome or ampulla cardiomyopathy) has recently been described. First recognised in Japanese patients, tako-tsubo refers to the end-systolic appearance of the left ventricle on ventriculography and its resemblance to the round bottomed, narrow necked Japanese fishing pots used to trap octopus

**Case presentation:**

We present two cases of female caucasian patients aged 40 and 63 years respectively admitted following severe stressful events who met the diagnostic criteria of tako-tsubo cardiomyopathy, namely acute chest pain, transient akinesis or dyskinesia of the left ventricle, new dynamic electrocardiogram changes and no significant epicardial coronary artery disease in the absence of recent head trauma, intracranial bleeding, phaeochromocytoma, myocarditis and hypertrophic cardiomyopathy. Both had elevated cardiac biomarkers. Characteristically the condition is transient and the abnormal akinesia/dyskinesia of the left ventricle has been observed to normalise within 1-month as in our patients who made full recoveries.

**Conclusion:**

Patients with tako-tsubo cardiomyopathy present with features consistent with an acute coronary syndrome and as such the syndrome is probably under-diagnosed. It may be with the introduction of primary percutaneous coronary intervention more cases are identified, sparing patients the risks of unnecessary thrombolytic therapy. Tako-tsubo cardiomyopathy should be considered in all patients presenting with acute onset chest pain and elevated cardiac biomarkers.

## Background

Tako-tsubo cardiomyopathy (idiopathic apical ballooning syndrome or ampulla cardiomyopathy) has recently been described. First recognised in Japanese patients, tako-tsubo refers to the end-systolic appearance of the left ventricle on ventriculography and its resemblance to the round bottomed, narrow necked Japanese fishing pots used to trap octopus [1]. The condition is characterised by chest pain with transient left ventricular (LV) systolic dysfunction frequently precipitated by a stressful event. We present two cases of Caucasian patients admitted following severe stressful events who met the diagnostic criteria of tako-tsubo cardiomyopathy, namely acute chest pain, transient akinesis or dyskinesia of the left ventricle, new dynamic ECG changes and no significant epicardial coronary artery disease (CAD) in the absence of recent head trauma, intracranial bleeding, phaeochromocytoma, myocarditis and hypertrophic cardiomyopathy [2].

## Case presentation

**Figure 1:**
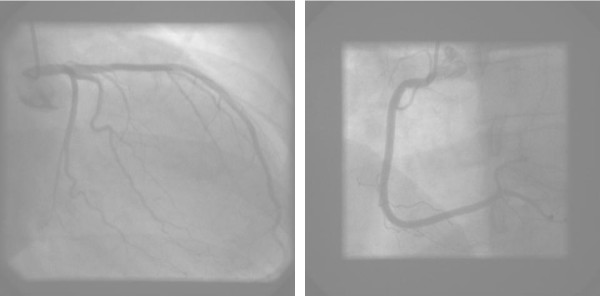
Diagnostic coronary angiography demonstrating normal epicardial coronary arteries.

A 40-year-old caucasian female presented with a 12-hour history of dyspnoea and intermittent chest discomfort at rest. The evening before she performed bystander CPR on her father who later died. The patient was on no regular medications and had no past medical history of note. Her cardiac risk factor profile included dyslipidaemia and a positive family history of CAD. She was a non-smoker with no history of hypertension or diabetes mellitus. Examination was unremarkable. Serial 12-lead ECG recordings demonstrated evolving antero-lateral T wave inversion. An ACS was diagnosed based on the history, ECG abnormalities and a raised troponin T (0.14 ng/ml, reference <0.01 ng/ml). She was commenced on aspirin, clopidogrel, low molecular weight heparin, beta-blocker, statin and ACE-inhibition. A transthoracic echocardiogram demonstrated severely impaired LV systolic function with akinesia of all mid- and apical segments, with normal basal contraction. Transfer to the regional cardiothoracic centre took place with diagnostic coronary angiography demonstrating normal epicardial coronary arteries (figure 1). A left ventriculogram confirmed impaired LV systolic function.

**Figure 2:**
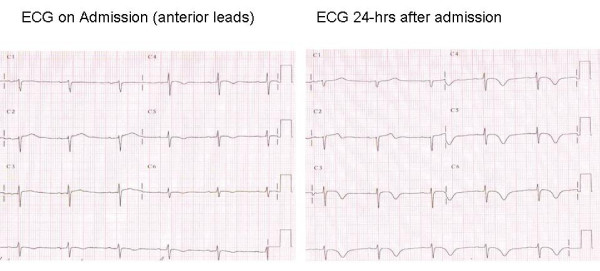
12 lead ECG showing evolution of widespread ST/T wave changes throughout the anterior cheat leads but no Q waves, consistent with tako-tsubo cardiomyopathy.

A 63-year-old caucasian female presented to her local emergency department with a 1-hour history of chest tightness and left arm pain after a confrontational argument with a neighbour where the patient was struck on the face. A 12-lead ECG showed lateral ST elevation. Her cardiac risk factor profile included dyslipidaemia and hypertension. There was no past medical history of note and she was a non-smoker. Due to the recent assault and facial bruising the patient was referred to the regional cardiothoracic centre for consideration of primary percutaneous coronary intervention rather than thrombolytic therapy. Diagnostic coronary angiography demonstrated non-obstructive coronary atheroma. Her ECGs evolved widespread anterior T wave inversion and her troponin T was elevated (0.54 ng/ml) (figure 2). A transthoracic echocardiogram showed mid/apical regional wall hypokinesia with moderately impaired LV systolic function.

In both cases the diagnosis was tako-tsubo cardiomyopathy. The patients conditions improved and they were discharged home on aspirin, statin, ACE-inhibition and beta-blocker therapy. They have both been reviewed in the outpatient clinic and are well. Repeat echocardiography has shown normalisation of LV systolic function.

## Discussion

Patients with tako-tsubo cardiomyopathy present with features consistent with an ACS and as such the syndrome is probably under-diagnosed. It may be with the introduction of primary percutaneous coronary intervention more cases are identified, sparing patients the risks of unnecessary thrombolytic therapy. Its prevalence in patients presenting with ACS ranges from 0.7–2.2% amongst both Japanese and western populations, although the true prevalence remains unknown [3]. There is a strong female predominance especially amongst post menopausal women [4]. Furthermore most patients present with a preceding history of extreme psychological and/or physical distress as in our two cases [3].

Our patients presented with the characteristic clinical features of the condition, acute onset chest pain and/or dyspnoea. In the acute phase pulmonary oedema and overt cardiogenic shock may occur in addition to fatal arrhythmias. The characteristic ECG features of tako-tsubo cardiomyopathy are non-specific and include dynamic ST elevation (usually less than in acute anterior myocardial infarction) and/or T wave inversion typically throughout the anterior leads [5]. Both of our patients also had marginally elevated cardiac biomarkers.

Transthoracic echocardiography will identify regional wall abnormalities, typically akinesia of the LV apex and/or the mid-portion of the LV. Typically the area of LV dysfunction usually involves a larger territory than that supplied by one epicardial coronary artery. Diagnostic coronary angiography needs to be performed in all patients to exclude obstructive epicardial CAD [2]. Ventriculography may demonstrate the apical ballooning that occurs in addition to hypercontraction of the basal segments. The condition does not always affect the apex and mid ventricular ballooning with sparing of the basal and apical segments may also occur. We did not perform cardiac MR (CMR) imaging or SPECT however CMR can provide information regarding functional involvement, chamber dimensions and presence of intramyocardial oedema and also allows the physician to exclude infarction and inflammatory processes [6].

The pathophysiology of the condition is debatable. Numerous hypotheses have been proposed, including diffuse coronary vasospasm, microvascular dysfunction, transient LV outflow tract obstruction, catecholamine mediated cardiotoxicity or cardiac autonomic imbalance [1,7]. Catecholamine mediated cardiotoxicity is the most widely proposed mechanism given that patients typically present with a preceding history of extreme psychological and/or physical distress implying increased sympathetic activity with a direct catecholamine toxic effect on the cardiac myocytes [8]. Results are however conflicting, some studies report elevated catecholamine levels in tako-tsubo cardiomyopathy whereas in others catecholamine levels have been normal [9]. It is more likely that the pathogenesis of the condition is mutlifactoral.

The overall prognosis of the condition is favourable with in hospital mortality rates of 1% and recurrence rates <3% [10]. However complications including cardiogenic shock and tachyarrhythmias can occur in up to 19% of patients [3,4]. As the condition causes LV impairment with apical ballooning the patients are also at a theoretical risk of LV thrombus formation and decisions regarding anticoagulation need to be made. Patients can also develop functional mitral regurgitation. Treatment relates to the patients haemodynamic status and no established guidelines exist [3]. Often treatment for an ACS is commenced. Both of our patients were treated with aspirin, beta-blockers and ACE-inhibition.

Characteristically the condition is transient and the abnormal akinesia/dyskinesia of the left ventricle has been observed to normalise within 1 month as in our patients who have both made full recoveries.

## Conclusion

Patients with tako-tsubo cardiomyopathy present with features consistent with an ACS and as such the syndrome is probably under-diagnosed. It may be with the introduction of primary percutaneous coronary intervention more cases are identified, sparing patients the risks of unnecessary thrombolytic therapy. Tako-tsubo cardiomyopathy should be considered in all patients presenting with acute onset chest pain and elevated cardiac biomarkers.

## Abbreviations

ACE: Angiotensin Converting Enzyme; ACS: Acute Coronary Syndrome; CAD: Coronary Artery Disease; CMR: Cardiac Magnetic Resonance; CPR: Cardio-Pulmonary Resuscitation; ECG: Electrocardiogram; LV: Left Ventricle; SPECT: Single Photon Emission Computed Tomography.

## Consent

Written informed consent was obtained from the patients for publication of this case report and accompanying images. A copy of the written consent forms is available for review by the Editor-in-Chief of this journal.

## Competing interests

The authors declare that they have no competing interests.

## Authors' contributions

AJT participated in the design, co-ordinated the image analysis and drafted the manuscript. RJG participated in the design and helped to draft the manuscript. JAH participated in the design and helped to draft the manuscript. All authors were involved in the clinical care and follow up of the patients outlined in the manuscript. All authors read and approved the final manuscript.
